# Notes and News

**Published:** 1903-11-21

**Authors:** 


### NOTES AND NEWS.

THE annual meeting of Presidents of the League of Mercy will be held in the Board Room of Charing Cross Hospital at 4 o'clock on December 18th.

THE late Mr. Richard Thomas, of Ealing, has bequeathed £1,000 to the Macclesfield Infirmary to endow a bed, and £200 to the Ealing Cottage Hospital. The late Mr. Carr Wigg, of Hove, has bequeathed £250 each to King's College Hospital, the Sussex County Hospital, and the Royal Alexandra Hospital, Brighton.

KING'S COLLEGE HOSPITAL is issuing an appeal for £300,000, signed by the Chairman of the Joint Committee on the removal of the hospital, and by the Chairman and Treasurer of the hospital. A site has been presented near Camberwell Green, on which it is hoped to build a thoroughly efficient and up-to-date hospital, for which, together with the expenses of removal, the sum appealed for will be required. Contributions may be sent to the Secretary.

FOR some time past a correspondence has been carried on between the joint Parliamentary Committee of the National Association, the Scottish Association, and the London Master Bakers' Protection Society and the Home Secretary in regard to the increase of cubic feet of air space for workers in bakehouses. The Incorporated Society of Medical Officers of Health recommended that the space should be increased from 250 cubic feet to a minimum of 500. The result is that the Home Secretary has informed the committee that a case has been made out in support of the medical officers' recommendations and that an order will be made for 500 cubic feet of air space per worker in underground bakehouses and for 400 cubic feet space in other cases where artificial other than electric light is used during working hours.

OWING to the conviction that obtains that rats are the means of conveying plague and other diseases, strenuous efforts are being made for their extermination at the Port of London. During October 6,000 were destroyed, and from the beginning of the year upwards of 60,000 have been destroyed. In 1902 the number was upwards of 185,900.

A SUCCESSFUL matinee was given at the Borough Theatre, Stratford, E., on the 12th instant, in aid of the Extension Fund of the West Ham Hospital. The theatre was lent for the occasion and the services of the theatre staff and of the performers were given gratuitously, so that the hospital will reap the gross results of the performance. This, however, will amount to but a trifle of what is sorely needed, since extension is imperatively called for, to meet the demands of a very poor and crowded neighbourhood. The hospital experiences great difficulty in raising support owing to its situation in a district far removed from the view of the wealthy.

ALDERMAN TRELOAR asks us to remind our generous readers of the distribution of Christmas hampers to upwards of six thousand poor crippled children in the metropolis. These welcome gifts are despatched direct from the Guildhall by the Lord Mayor and Sheriffs on the morning of the day when, by permission of the Corporation, 1,200 Ragged School children are entertained. All through the year the work by the Ragged School Union of registration, visitation and relief continues, or it would not be possible to undertake the hamper distribution on such a scale, with the certainty that not one goes astray or falls into undeserving hands. The little cripple entertains the family, and on a modest estimate the fund brightened the lives last Christmas of over thirty thousand of the poor of London. Donations may be sent, as heretofore, addressed to Alderman Treloar, Little Cripples' Christmas Hamper Fund, 69 Ludgate Hill, London, E.C.

THE Assistance Publique was charged more than a year ago with the much-needed reconstruction of the Paris hospitals by the Municipal Council and to raise a loan of 40 million francs. The work of reconstruction is to be commenced next spring, and in the meantime a special committee are visiting the principal hospitals on the Continent with the object of obtaining information as to the plan of construction and the working of these various institutions. The members of this committee have just returned from a visit to Antwerp and Liège. At the former place the well-known Sturvenberg Hospital was visited. The institution has 350 beds, and is fitted with all the latest improvements, but the architecture is the most interesting portion. The buildings are constructed in the shape of a star with eight arms all converging on to a central building, which includes the chapel and provides accommodation for the medical and administrative staffs, etc. At Liège the committee visited a less pretentious hospital founded six centuries ago by the prince-bishops of Bavaria, but it has recently been reconstructed, and on that account was worth a visit. The committee have expressed themselves as highly pleased with all they saw, and their observations will be the subject of a report.

